# Lactate-functionalized 3D-printed PCL/nHA scaffold drives BMSC osteogenesis via metabolic-epigenetic crosstalk

**DOI:** 10.1016/j.mtbio.2025.102101

**Published:** 2025-07-16

**Authors:** Min Zeng, Hao Liu, Wei Lu, Can Chen, Zhangyuan Lin, Ruibo Zhao

**Affiliations:** a Department of Orthopedics, Xiangya Hospital, Central South University, Changsha, China; b National Clinical Medical Research Center of Geriatric Diseases, Xiangya Hospital, Central South University, Changsha, China

**Keywords:** 3D-printed scaffold, Sodium lactate, Metabolic-epigenetic crosstalk, Lysine lactylation, Bone regeneration

## Abstract

Critical-sized bone defects pose significant clinical challenges due to the limited regenerative potential of human bone mesenchymal stem cells (BMSCs). To address this, we developed a 3D-printed polycaprolactone/nano-hydroxyapatite scaffold functionalized with sodium lactate (PCL/nHA/SL) to synergistically integrate structural support with metabolic-epigenetic modulation. The lactate-functionalized scaffold demonstrated excellent biocompatibility, facilitating BMSC adhesion, proliferation, and osteogenic differentiation. Compared to non-functionalized controls, the PCL/nHA/SL scaffold markedly enhanced osteogenesis, as evidenced by accelerated mineralization and upregulation of key osteogenic markers. Proteomic analysis revealed that lactate incorporation induced lysine lactylation modifications, with STAT1 identified as a central regulatory target. Mechanistic studies established that lactylation redirected STAT1 subcellular localization, thereby liberating RUNX2 to activate osteogenic transcriptional programs. Genetic validation underscored the critical role of STAT1 lactylation in orchestrating this metabolic-epigenetic crosstalk. *In vivo* evaluations further demonstrated the scaffold's capacity to drive functional bone regeneration in critical-sized defects, achieving robust trabecular bone formation. This study introduces a novel biomaterial strategy that couples 3D-printed architecture with lactate-driven metabolic reprogramming to overcome intrinsic barriers in BMSC-mediated osteogenesis. The findings highlight the potential of metabolic-epigenetic engineering in bone tissue regeneration and provide a translatable platform for complex defect repair.

## Graphical abstract

This illustration depicts a 3D-printed PCL/nHA scaffold releasing lactate to drive BMSC metabolic reprogramming and STAT1 lactylation. Cytoplasmic STAT1 relocation activates nuclear Runx2, triggering osteogenic gene expression. In vivo, the PCL/nHA/SL scaffold regenerates critical-sized bone defects with mature trabeculae.

**Figure:**
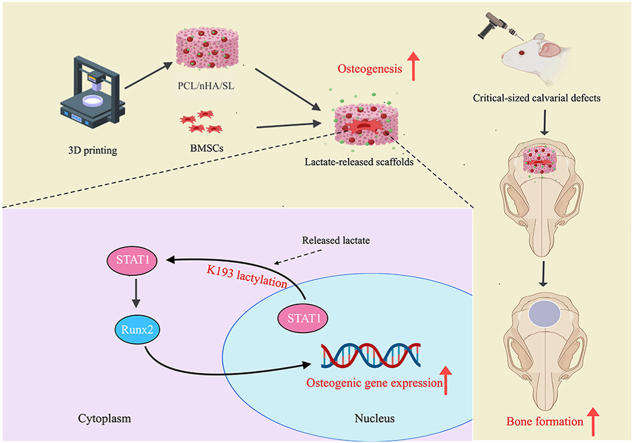

## 1 Introduction

Critical-sized bone defects caused by trauma, infection, or tumor resection present persistent clinical challenges. Current gold-standard treatments such as autografts and allografts are constrained by donor scarcity, immune complications, and infection risks, driving the need for synthetic alternatives [[1], [2], [3]]. Tissue engineering strategies combining biomimetic scaffolds with stem cells hold therapeutic potential, yet achieving sustained osteogenic induction while maintaining metabolic compatibility with host microenvironments remains an unmet goal [4,5].

Polycaprolactone (PCL)-based scaffolds are prominent in bone regeneration due to their tunable biodegradation, mechanical durability, and regulatory approval [6,7]. However, PCL's inherent hydrophobicity and limited bioactivity require composite strategies with bioactive components. Nano-hydroxyapatite (nHA)—the primary inorganic constituent of natural bone—enhances osteoconductivity [8,9], but existing designs fail to replicate the dynamic metabolic interplay of native bone tissue. Human mesenchymal stem cells (MSCs), particularly bone marrow-derived populations (BMSCs), are central to skeletal repair through multipotency and paracrine functions [10]. Conventional normoxic in vitro expansion compromises MSC stemness and osteogenic capacity, whereas the hypoxic bone marrow niche preserves these properties via metabolic adaptations [11], suggesting scaffold-mediated metabolic modulation could improve regenerative outcomes.

Lactate exhibits cell-type-dependent duality in bone biology. It exerts pro-osteogenic effects in BMSCs via histone H3K18la lactylation [12] or olfactory receptor Olfr1440 activation [13], yet inhibits osteogenesis in periodontal ligament stem cells through MCT1-mTOR-mediated autophagy suppression [14]. Concurrently, lactate drives immunomodulatory reprogramming, polarizing macrophages toward M2 phenotypes, and suppressing neutrophil mobilization through GPR81 signaling [15,16]. While physiological lactate accumulation in fracture sites enhances vascularization and osteoblast activation, aberrant lactylation disrupts immune regulation [17,18]. This functional pleiotropy underscores the need for spatially controlled lactate delivery. Sodium lactate (SL) incorporation offers a strategic approach to locally modulate lactate levels, mimicking hypoxic niches. Although lactate-enriched microenvironments elevate osteogenic markers [13], mechanisms linking scaffold-derived lactate to epigenetic osteogenesis regulation remain unresolved.

Despite advances, critical gaps persist: 1) No scaffold designs exploit lactate's pro-osteogenic potential; 2) Mechanisms beyond histone lactylation remain unexplored; and 3) Lactate's impact on master regulators like RUNX2 is unknown. To address these, we engineer a 3D-printed PCL/nHA scaffold functionalized with sodium lactate (**PCL/nHA/SL**). We pioneer a novel mechanism wherein scaffold-released lactate induces **STAT1 lactylation**-distinct from histone-focused pathways [12,19]-disrupting STAT1-RUNX2 binding to derepress osteogenesis. Through multi-omics analyses and genetic validation, we elucidate the metabolic-epigenetic crosstalk. *In vivo,* PCL/nHA/SL repairs critical-sized defects, establishing a biomaterial paradigm integrating structural and biochemical modulation.

## 2 Methods

### 2.1 Scaffold fabrication and characterization

Composite scaffolds of PCL (Sigma-Aldrich), nHA (Sigma-Aldrich), and SL (Sigma-Aldrich) were fabricated using a solvent casting and 3D printing approach. PCL pellets (70 % w/w) and nHA (30 % w/w) were dissolved in a 1:1 (v/v) mixture of tetrahydrofuran and dimethylformamide under magnetic stirring (40 °C, 24 h), and similar material proportion have previously been reported in the literature for preparation of PCL/nHA composite materials [6,20,21]. For PCL/nHA/SL scaffolds, varying lactate concentrations (0.5 %–5 %) of SL were added to the homogeneous solution. The mixture was loaded into a custom extrusion-based 3D printer (Regenovo) equipped with a 200-μm nozzle. Scaffolds were printed with a layer-by-layer architecture (fiber diameter: 200 ± 50 μm; pore size: 330 ± 50 μm) and post-cured at 60 °C for 12 h. Cylindrical scaffolds (Φ = 5 mm, height = 2 mm) were sterilized using ^60^Co γ-irradiation (25 kGy) and rinsed twice with sterile PBS prior to cell seeding. The pH value, weight loss, and Ca^2+^ release over time from scaffolds were measured in triplicate after 1, 3, 7, 14, 21, and 28 days of immersion. Scaffolds were immersed in simulated body fluid (SBF) at 37 °C with constant shaking for 28 days to assess the degradation rate in vitro. The ion concentrations of the scaffolds were measured by inductively coupled plasma optical emission spectrometry (ICP-OES, Thermo Fisher, USA), and the pH values were detected with a pH meter (Beckman Instruments, USA) at the predetermined time points.

### 2.2 Isolation and culture of BMSCs

Human BMSCs (ATCC® PCS-500-012™) were expanded in Dulbecco's Modified Eagle Medium (DMEM; Gibco) supplemented with 10 % fetal bovine serum (FBS; Biological Industries) and 1 % penicillin/streptomycin (Solarbio) at 37 °C in a 5 % CO_2_ humidified incubator. Cells at passage 3 were used for experiments. For scaffold seeding, 4 × 10^4^ cells per scaffold were inoculated onto sterilized scaffolds in 24-well plates and cultured in either basal medium or osteogenic induction medium (OM: DMEM supplemented with 0.1 mM dexamethasone, 8 mM β-glycerophosphate, and 50 μg/mL ascorbic acid). Medium was refreshed every 3 days.

### 2.3 Biocompatibility and cell viability assays

Cell viability was assessed using a LIVE/DEAD® Viability/Cytotoxicity Kit (BestBio). Scaffolds were incubated with 2 μM calcein-AM and 4 μM propidium iodide at 37 °C for 30 min, followed by imaging using a Cytation5 cell imaging reader (BioTek). Proliferation was quantified via CCK-8 (Abbkine) at 1, 3, 5, and 7 days. Absorbance (450 nm) was measured using a microplate reader (BioTek). For cytoskeletal analysis, cells were fixed with 4 % paraformaldehyde (PFA), permeabilized with 0.1 % Triton X-100, and stained with Alexa Fluor™ 594-phalloidin (Thermo Fisher) and DAPI. Images were acquired using a Zeiss LSM 880 confocal microscope (63 × oil immersion lens).

### 2.4 Osteogenic differentiation assessment

Alkaline phosphatase (ALP) activity was measured at days 7 and 14 using a p-nitrophenyl phosphate (pNPP) substrate (Beyotime). Lysates were centrifuged (6000 rpm, 10 min), and supernatants were incubated with pNPP at 37 °C for 30 min. Absorbance at 405 nm was normalized to total protein concentration (BCA assay). Calcium deposition was evaluated using xylenol orange (XO, 20 μM) and Alizarin Red S (ARS) staining. Fluorescence intensity (Texas Red filter) and mineralized nodule area were quantified via ImageJ. Osteogenic markers (RUNX2, COL1A1, OCN, OPN) were analyzed by western blot (20 μg protein/lane) using specific antibodies (Abcam, CST; dilution 1:1000). Lactate levels in culture medium were measured at days 1, 7, 14, and 21 using a Lactate Assay Kit (Biovision, K607-100).

### 2.5 Lysine lactylation proteomics

BMSCs were cultured on PCL/nHA/SL or PCL/nHA scaffolds for 14 days. Proteins were extracted, digested with trypsin, and lactylated peptides were enriched using anti-K-Lac antibody-conjugated beads (PTM Bio). Peptides were analyzed by 4D label-free LC-MS/MS on a timsTOF Pro mass spectrometer (Bruker) with PASEF mode. Data were processed using MaxQuant (v1.6.15.0) against the UniProt human database (2022). Differentially lactylated sites (FC > 1.5, P < 0.05) were identified, followed by GO/KEGG enrichment (Fisher's exact test, P < 0.05). STAT1-K193 lactylation was validated by manual inspection of MS/MS spectra (Mascot score >200, localization probability >99 %).

### 2.6 Genetic modification and experimental groups

In these experiments, BMSCs were cultured with no osteogenic induction. Lentiviral transduction (MOI = 20, 72h incubation with 2 μg/mL polybrene) was performed for genetic modifications. Groups were designed to dissect STAT1 lactylation-dependent regulation of Runx2 (Table 1).

**Table 1:** STAT1 lactylation disrupts STAT1-Runx2 interaction.

Groups	Detailed experimental setup
Scaffold-free BMSCs (NC vector)	Scaffold-free BMSCs transduced with empty vector (baseline control)
Scaffold-free BMSCs (Runx2-OE)	Scaffold-free BMSCs with Runx2 overexpression (pLVX-Runx2)
Scaffold-free BMSCs (Runx2-OE + STAT1-KD)	Scaffold-free BMSCs with Runx2-OE and STAT1 knockdown (shSTAT1)
Scaffold-free BMSCs (Runx2-OE + STAT1-OE)	Scaffold-free BMSCs with Runx2-OE and wild-type STAT1 overexpression (pLVX-STAT1-WT)
Scaffold-containing BMSCs (Runx2-OE + STAT1-OE)	BMSCs with Runx2-OE and wild-type STAT1 overexpression (pLVX-STAT1-WT) cultured on PCL/nHA/SL scaffold

Mechanistic assays were performed 48h post-transfection. STAT1/Runx2 co-localization was assessed by immunofluorescence using anti-STAT1 and anti-Runx2 antibodies. Co-immunoprecipitation used STAT1 antibody for pull-down, with IgG control; blots probed for lactylation, Runx2, and inputs. Chromatin immunoprecipitation quantified Runx2 binding to the osteocalcin promoter using anti-Runx2 antibody with IgG control. Western blots detected Runx2 and osteocalcin expression. All assays included GAPDH normalization.

BMSCs were transduced with shSTAT1 lentivirus to knockdown endogenous STAT1. Cells were rescued with HA-tagged STAT1-WT or lactylation-deficient STAT1-K193R lentivirus. These cell lines (STAT1-KD + STAT1-WT or STAT1-KD + STAT1-K193R) were seeded onto PCL/nHA/SL scaffolds and cultured in osteogenic medium for 14 days (Table 2).

**Table 2:** STAT1-K193R abrogates lactate-induced osteogenesis.

Groups	Detailed experimental setup
Scaffold-free BMSCs (NC vector)	BMSCs without scaffold (negative control)
Scaffold-containing BMSCs (NC vector)	BMSCs on PCL/nHA/SL scaffold (positive control)
Scaffold-containing BMSCs (STAT1-KD)	STAT1-knockdown BMSCs loaded on PCL/nHA/SL scaffold
Scaffold-containing BMSCs (STAT1-KD + STAT1-WT)	STAT1-knockdown BMSCs rescued with STAT1-WT loaded on PCL/nHA/SL scaffold
Scaffold-containing BMSCs (STAT1-KD + STAT1-K193R)	STAT1-knockdown BMSCs rescued with lactylation-deficient STAT1-K193R mutant loaded on PCL/nHA/SL scaffold

Osteogenic capacity was assessed by: alkaline phosphatase activity, alizarin red S mineralization and Western blotting for Runx2, COL1A1, OCN, and OPN (Day 14). Lactate levels were quantified throughout.

### 2.7 STAT1 lactylation and functional studies

For co-immunoprecipitation (Co-IP), cell lysates were incubated with anti-STAT1 antibody (Proteintech) or control IgG overnight at 4 °C, followed by Protein A/G agarose pulldown. Precipitates were immunoblotted with anti-K-lactylation (PTM Bio, 1:300) and anti-STAT1 (1:500). Subcellular localization of STAT1 was assessed via immunofluorescence (IF) using anti-STAT1 (1:200) and Alexa Fluor® 488/555 secondary antibodies. HA-tagged STAT1 mutants (K193R, K584R) were transfected into 293T cells using Lipofectamine 3000. STAT1-RUNX2 interaction was evaluated by chromatin immunoprecipitation (ChIP) with anti-RUNX2 antibody and osteocalcin promoter-specific primers.

### 2.8 *In vivo* bone regeneration

Critical-sized calvarial defects (5 mm diameter) were created in Sprague-Dawley rats (n = 6/group). Scaffolds were implanted into defects, while controls remained untreated. After 12 weeks, samples were harvested, fixed in 4 % PFA, and analyzed via micro-CT (Skyscan 1176). Histological sections were stained with hematoxylin/eosin (H&E), Masson's trichrome, and Immunohistochemical (IHC) staining (OCN and RUNX2). New bone formation was quantified (BV/TV, Tb.N, and Tb.Th) using CTAn software (Bruker).

### 2.9 Statistical analysis

Data are presented as mean ± SD (n = 3). Differences were assessed using Student's t-test or one-way ANOVA with Tukey's post hoc test (GraphPad Prism 9.0). Significance was set at *p* < 0.05.

## 3 Results

### 3.1 Scaffold biocompatibility and optimal lactate concentration determination

**Fig. 1:**
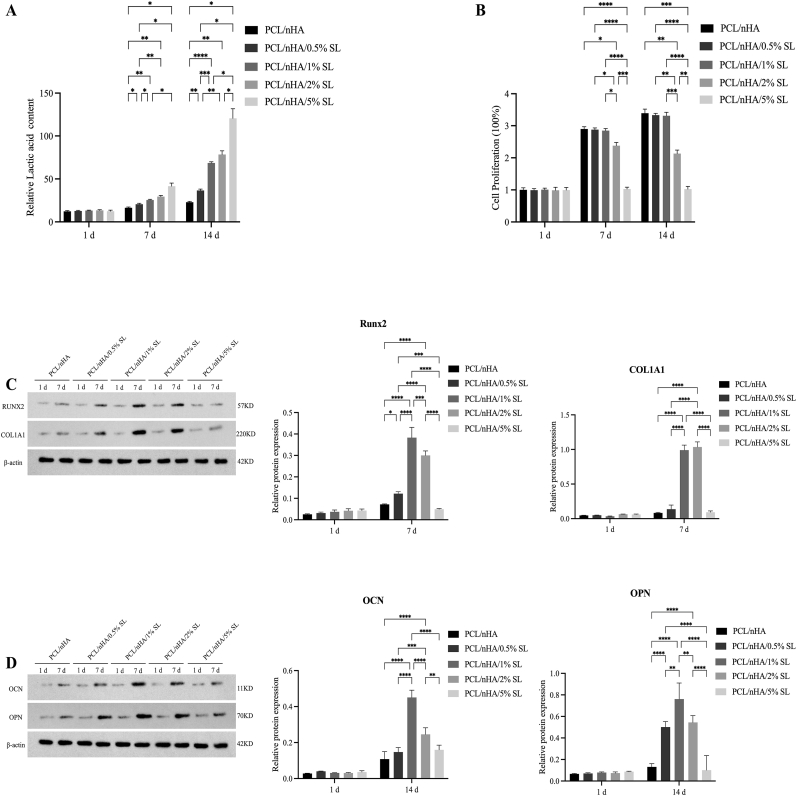
**Optimal lactate concentration determination in** PCL/nHA/SL scaffolds. (A) Lactate release kinetics showing sustained efflux from PCL/nHA/SL scaffolds versus baseline levels in PCL/nHA. (B) CCK-8 assays showed 0.5 %–1 % (w/w) lactate maintained cell viability, while 5 % significantly suppressed proliferation. (C) Western blot analysis of early osteogenic markers (Runx2, COL1A1) during 7-day culture. (D) Western blot analysis of later osteogenic markers (OCN, OPN) during 14-day culture. (Data normalized to β-actin; Data expressed as mean ± SD; ∗*p* < 0.05, ∗*∗p* < 0.01, ∗∗∗*p* < 0.001, ∗∗∗∗*p* < 0.0001).

BMSCs cultured on PCL/nHA scaffolds with lactate concentrations (0.5 %–5 % w/w) exhibited dose-dependent responses. CCK-8 assays confirmed that 0.5 %–1 % lactate maintained cell viability, whereas 5 % significantly suppressed proliferation (*p* < 0.05 vs. control). Western blotting demonstrated optimal osteogenic differentiation at 1 % lactate, with 7-day RUNX2 (5.3-fold) and COL1A1 (12.0-fold) upregulation and 14-day OCN (4.2-fold) and OPN (5.8-fold) elevation (*p* < 0.001 vs. control). High lactate (5 %) impaired both viability and differentiation (Fig. 1). Based on these findings, 1 % lactate was selected for subsequent studies.

**Fig. 2:**
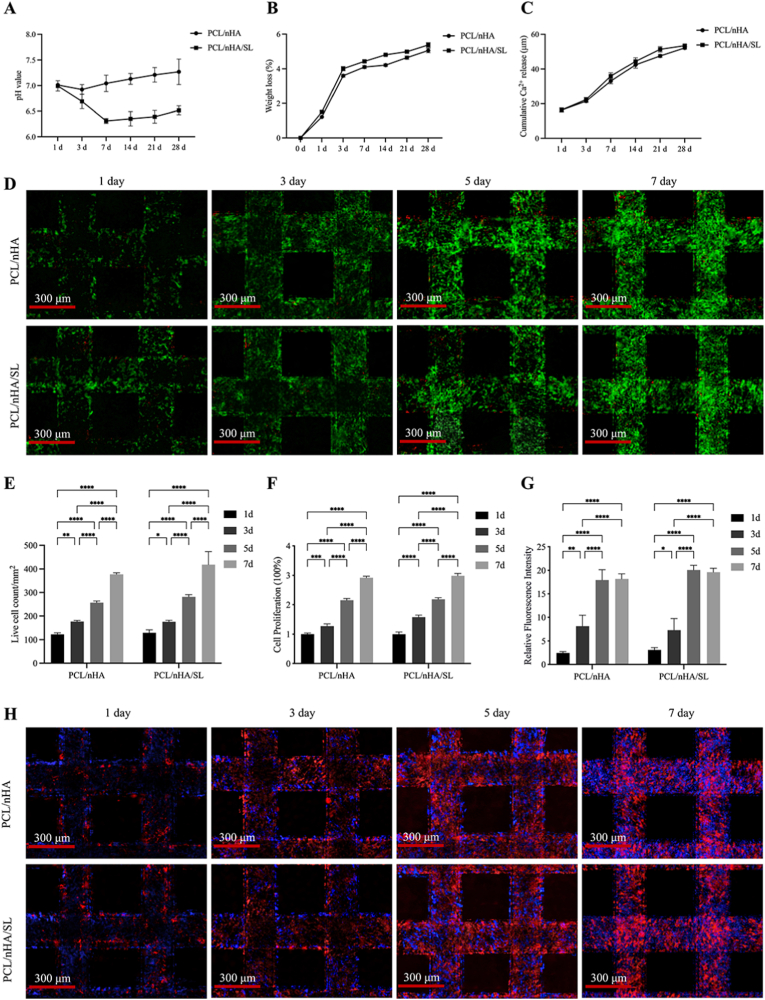
Biocompatibility evaluation of PCL/nHA and PCL/nHA/SL composite scaffolds. (A)pH value of the SBF. (B) The weight loss of the scaffolds in the SBF. (C) Ca^2+^ release over time from scaffolds. (D) Live/dead staining visualization of BMSCs cultured on scaffolds at days 1, 3, 5, and 7 (Scale bars: 300 μm; viable cells: green; dead cells: red). (E) Quantitative analysis of live cell density showing comparable proliferation kinetics between groups. (F) CCK-8 assay confirming time-dependent cell proliferation without intergroup differences (p > 0.05). (G, H) Representative phalloidin/DAPI staining and quantification of fluorescence intensity demonstrating extensive cytoskeletal spreading (Scale bars: 300 μm; F-actin: red; nuclei: blue). (Data expressed as mean ± SD; ∗*p* < 0.05, ∗*∗p* < 0.01, ∗∗∗*p* < 0.001, ∗∗∗∗*p* < 0.0001). (For interpretation of the references to colour in this figure legend, the reader is referred to the Web version of this article.)

Throughout 28-day degradation, PCL/nHA/SL scaffolds maintained slight acidity (pH 5.8–6.6) with sustained low-concentration Ca^2+^ release (Fig. 2A–C). Biocompatibility was validated by >95 % viability (live/dead staining), comparable proliferation (CCK-8; *p* > 0.05), and extensive cytoskeletal spreading (phalloidin) on both scaffolds (Fig. 2D–H).

### 3.2 Temporal dynamics of osteogenic differentiation

**Fig. 3:**
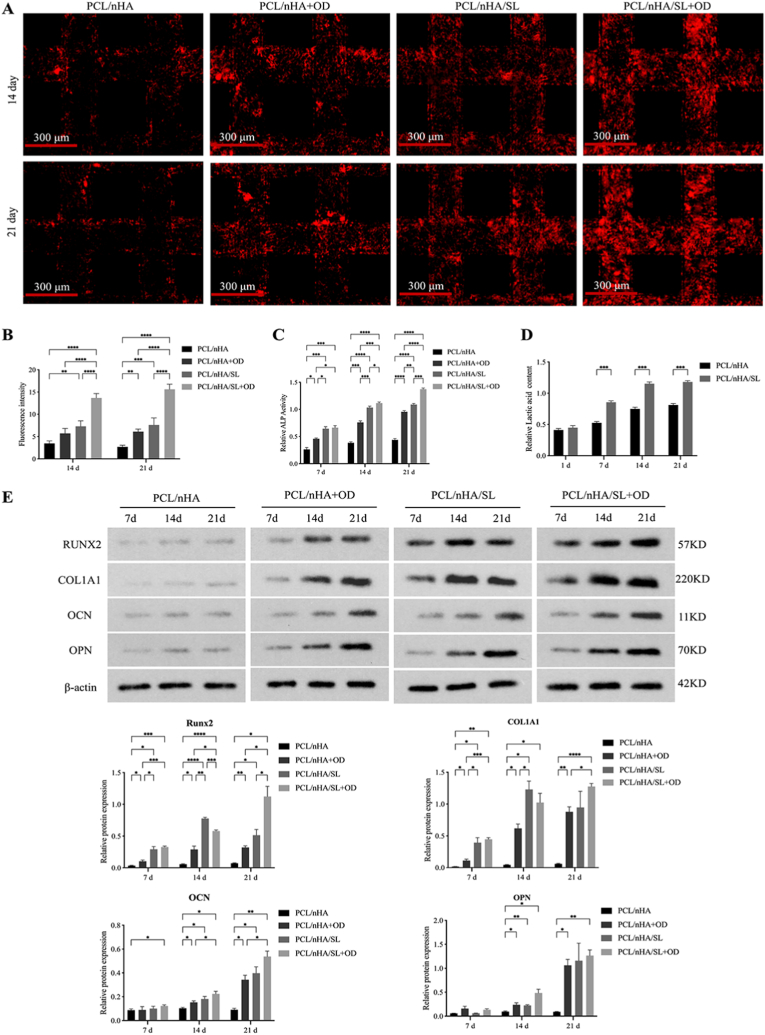
Osteogenic differentiation potential of scaffolds. (A) XO staining (Day 14 and 21) showing calcium deposition (red fluorescence, Scale bars: 300 μm). (B) Quantification of mineralization area (%). (C) Temporal ALP activity profiles: PCL/nHA/SL scaffolds enhanced early-stage differentiation, while combinatorial treatment (PCL/nHA/SL + osteogenic induction) achieved peak activity. (D) Lactate release kinetics showing sustained efflux from PCL/nHA/SL scaffolds versus baseline levels in PCL/nHA. (E) Western blot analysis of osteogenic markers (Runx2, COL1A1, OCN, OPN) during 21-day culture. (Data normalized to β-actin; Data expressed as mean ± SD; ∗*p* < 0.05, ∗*∗p* < 0.01, ∗∗∗*p* < 0.001, ∗∗∗∗*p* < 0.0001). (For interpretation of the references to colour in this figure legend, the reader is referred to the Web version of this article.)

Using the optimized 1 % SL concentration, we evaluated scaffold osteoinductive capacity. Under basal conditions, PCL/nHA/SL scaffolds demonstrated intrinsic mineralization activity with 2.1-fold higher calcium deposition than PCL/nHA controls at day 14 (7.28 ± 1.24 vs. 3.46 ± 0.56; *p* < 0.01; Fig. 3A and B). This advantage amplified under osteogenic induction (OD), where PCL/nHA/SL + OD achieved 2.4-fold greater mineralization than PCL/nHA + OD by day 21 (13.66 ± 1.01 vs. 5.71 ± 1.12; *p* < 0.0001). ALP activity analysis revealed SL-mediated acceleration of early differentiation, showing 2.4–2.7-fold increases from day 7 onward (*p* < 0.001; Fig. 3C), with synergistic PCL/nHA/SL + OD treatment producing peak activity (3.1-fold vs. controls; *p* < 0.0001) at day 21. Parallel lactate release profiling confirmed sustained efflux from PCL/nHA/SL scaffolds (Fig. 3D), indicating degradation dynamically regulates local lactate availability.

Stage-specific marker analysis further delineated temporal progression: At day 7, SL-dependent upregulation occurred in early markers Runx2 (8.4-fold; *p* < 0.05) and COL1A1 (23.5-fold; *p* < 0.05). By day 14, this extended to enhanced Runx2 (13.4-fold; *p* < 0.0001), COL1A1 (27.9-fold; *p* < 0.05), and late markers OCN (1.8-fold) and OPN (2.3-fold; both *p* < 0.05). At day 21, PCL/nHA/SL + OD exhibited amplified terminal marker expression: Runx2 (15.5-fold), COL1A1 (20.9-fold), OCN (5.9-fold), and OPN (14.0-fold) (all *p* < 0.05 vs. controls; Fig. 3E). These molecular dynamics directly paralleled biomineralization patterns, confirming a coordinated multi-stage osteogenic enhancement mechanism.

### 3.3 Lactylation-mediated epigenetic regulation

**Fig. 4:**
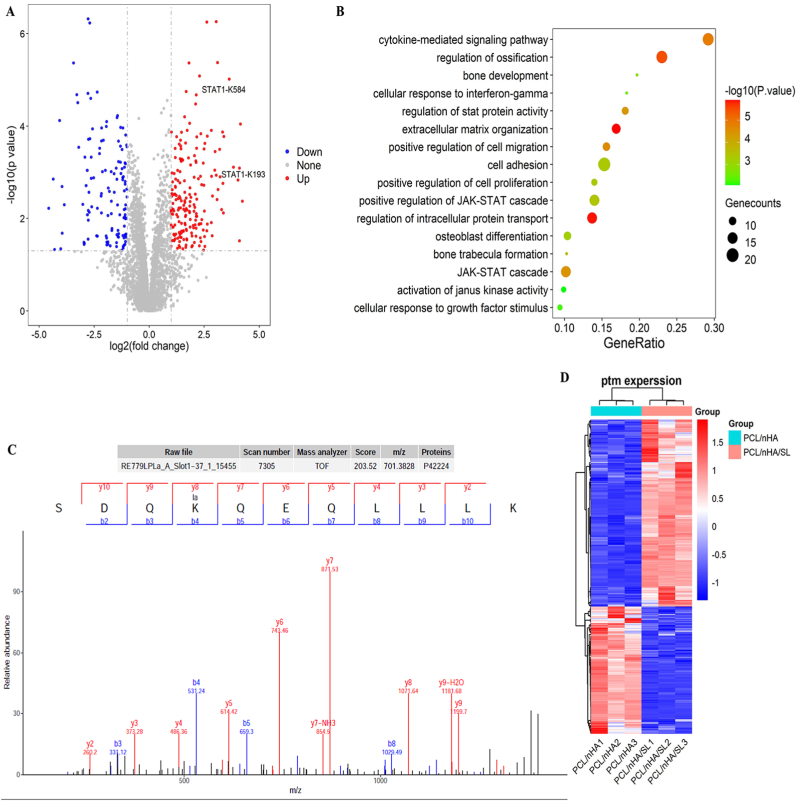
Lysine lactylation proteomics. (A) Volcano plot of lactylation sites (PCL/nHA vs. PCL/nHA/SL). Red/blue: up/down regulated sites. (B) GO/KEGG enrichment of lactylated proteins (e.g., regulation of ossification, osteoblast differentiation, and JAK-STAT cascade). (C) MS/MS spectrum validating STAT1-K193 lactylation. (D) Sequence motif analysis of lactylation sites. Heatmap shows amino acid preference around lactylation sites. (For interpretation of the references to colour in this figure legend, the reader is referred to the Web version of this article.)

Given lactate's role in differentiation, 4D proteomics mapped lysine lactylation (K-Lac) modifications (Fig. 4). Quantitative profiling identified 376 differentially lactylated sites across 190 proteins in PCL/nHA/SL-cultured BMSCs versus controls, including upregulated sites on STAT1. Functional enrichment linked these modifications to bone formation and JAK-STAT signaling. MS/MS validation confirmed STAT1-K193 lactylation, implicating STAT1 as a lactate-osteogenesis mediator.

### 3.4 Functional characterization of STAT1 lactylation

**Fig. 5:**
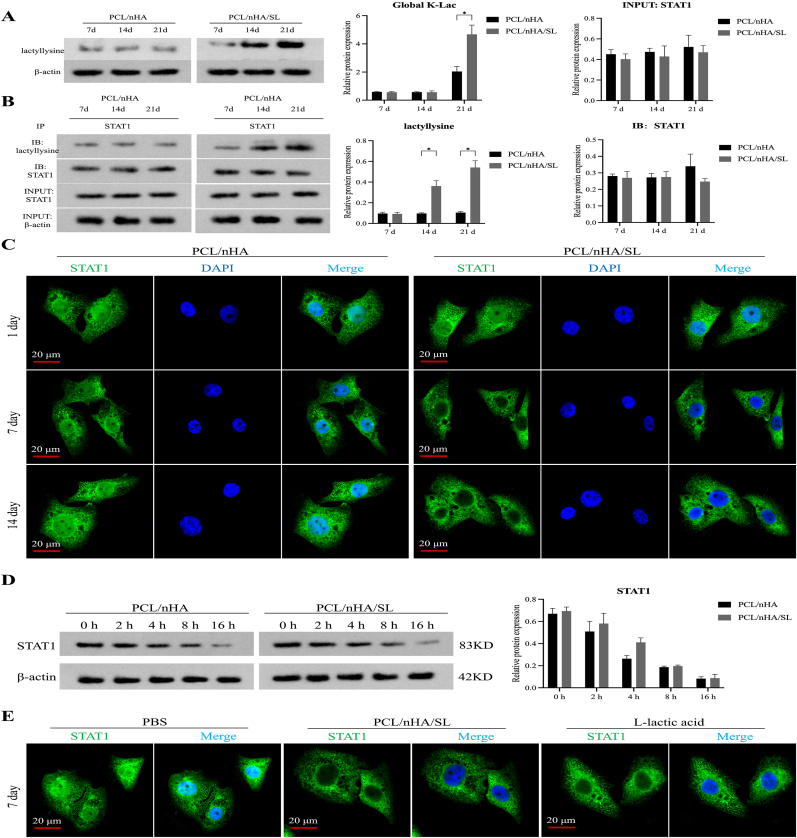
Functional characterization of STAT1 lactylation**.** (A) Global K-Lac levels detected by immunoblotting. (B) STAT1-lactylation confirmed by Co-IP with anti-lactyllysine antibody. (C) Subcellular STAT1 localization: cytoplasmic accumulation in PCL/nHA/SL group (Days 7–14) vs. nuclear-cytoplasmic distribution in PCL/nHA controls (Scale bars: 20 μm; STAT1: green, DAPI: blue). (D) STAT1 protein stability assessed by cycloheximide chase assay. (E) Lactate-induced STAT1 cytoplasmic translocation by immunofluorescence. (Data normalized to β-actin; Data expressed as mean ± SD; ∗*p* < 0.05, ∗*∗p* < 0.01, ∗∗∗*p* < 0.001, ∗∗∗∗*p* < 0.0001). (For interpretation of the references to colour in this figure legend, the reader is referred to the Web version of this article.)

Global lysine lactylation increased time-dependently in PCL/nHA/SL scaffolds, peaking at 4.3-fold versus controls at day 21 (*p* < 0.05; Fig. 5A). Co-IP confirmed STAT1 lactylation elevation from day 7 (3.7-fold; *p* < 0.05) to day 14 (5.2-fold; *p* < 0.05; Fig. 5B), with unaltered STAT1 protein levels (*p* > 0.05). Immunofluorescence revealed lactate-induced cytoplasmic STAT1 accumulation (Fig. 5C), while cycloheximide assays showed lactylation extended STAT1 half-life from 4.94 h to 3.07 h (Fig. 5D). Direct lactate stimulation recapitulated cytoplasmic translocation (Fig. 5E), mechanistically linking lactate release to STAT1 redistribution.

### 3.5 K193 lactylation governs STAT1 function

**Fig. 6:**
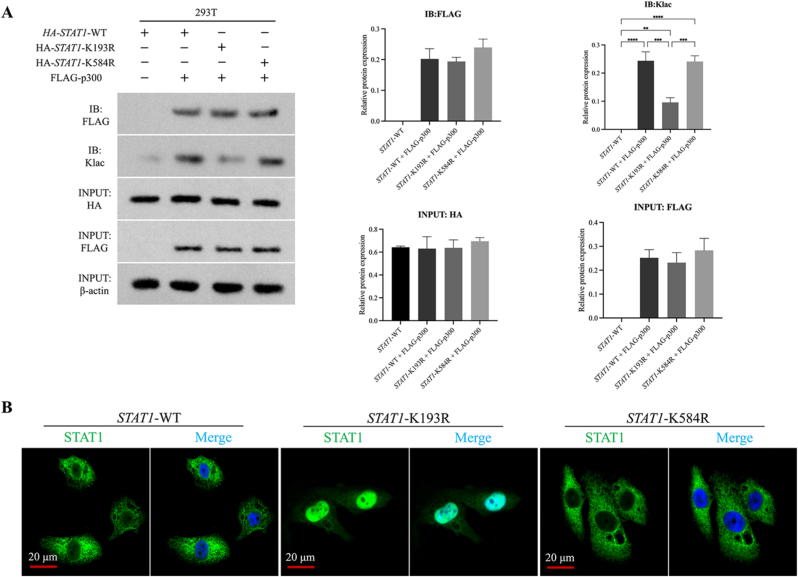
K193 lactylation governs STAT1 subcellular trafficking. (A) Co-IP validation of differential lactylation levels in STAT1 mutants. (B) Subcellular localization of HA-tagged STAT1 variants: WT and K584R localized to cytoplasm in PCL/nHA/SL cultures, whereas K193R retained nuclear localization (Scale bars: 20 μm; STAT1: green; DAPI: blue). (Data normalized to β-actin; Data expressed as mean ± SD; ∗*p* < 0.05). (For interpretation of the references to colour in this figure legend, the reader is referred to the Web version of this article.)

To identify the critical lactylation site regulating STAT1 function, we performed site-directed mutagenesis in 293T cells co-transfected with HA-tagged STAT1 (WT, K193R, K584R) and FLAG-p300. Co-IP (Fig. 6A) confirmed comparable physical interaction between all STAT1 variants and p300 (WT: 0.202 ± 0.033; K193R: 0.194 ± 0.013; K584R: 0.239 ± 0.027; *p* > 0.05). Lactylation-specific immunoblotting, however, revealed that the K193R mutation reduced STAT1 lactylation by 60.7 % (0.096 ± 0.017 vs. WT: 0.244 ± 0.032; *p* < 0.0001), whereas K584R had no effect (0.241 ± 0.020; *p* > 0.05). Input controls confirmed equivalent STAT1 expression (WT: 0.631 ± 0.105; K193R: 0.637 ± 0.070; K584R: 0.695 ± 0.032; *p* > 0.05), and FLAG-p300 was detected only in co-transfected samples. Functional validation in BMSCs (Fig. 6B) showed that both HA-STAT1(WT) and HA-STAT1(K584R) exhibited cytoplasmic localization in PCL/nHA/SL cultures. In contrast, HA-STAT1(K193R) remained nuclear, demonstrating that K193 lactylation is essential for STAT1 nuclear export. These results identify K193 as the key lactylation site modulating STAT1 subcellular dynamics.

### 3.6 Lactylation disrupts STAT1-Runx2 interaction

**Fig. 7:**
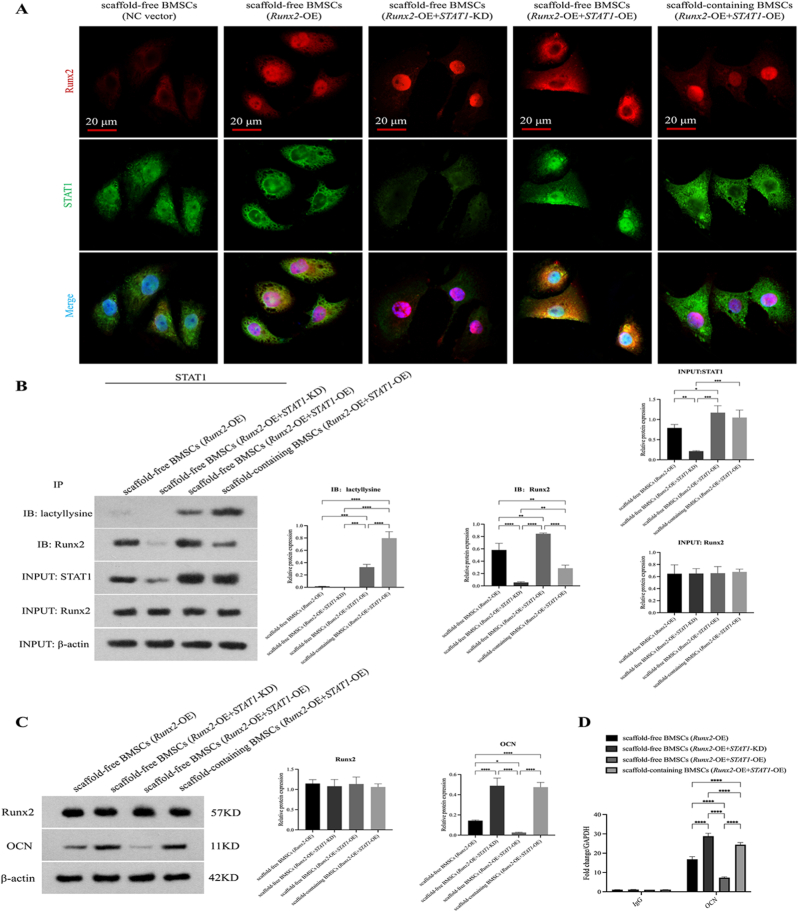
Lactylation-dependent STAT1-Runx2 interaction. (A) Confocal imaging of Runx2 nuclear translocation: Enhanced in STAT1-KD cells, suppressed by STAT1-OE, and rescued by scaffold co-culture (Scale bars: 20 μm; Runx2: red; STAT1: green; DAPI: blue). (B) Co-IP analysis of STAT1-Runx2 binding. (C) OCN expression modulated by STAT1-Runx2 axis. (D) ChIP-qPCR showing lactylation-dependent STAT1 chromatin occupancy at Runx2 promoter. (Data normalized to β-actin; Data expressed as mean ± SD; ∗*p* < 0.05, ∗*∗p* < 0.01, ∗∗∗*p* < 0.001, ∗∗∗∗*p* < 0.0001). (For interpretation of the references to colour in this figure legend, the reader is referred to the Web version of this article.)

To delineate how STAT1 lactylation regulates Runx2-driven osteogenesis, experiments utilized five groups: Scaffold-free BMSCs (NC vector, Runx2-OE, Runx2-OE + STAT1-KD, Runx2-OE + STAT1-OE) and Scaffold-containing BMSCs (Runx2-OE + STAT1-OE). Immunofluorescence (Fig. 7A) showed enhanced Runx2 nuclear translocation in STAT1-KD cells, while STAT1-OE caused cytoplasmic retention. This retention was reversed in scaffold-containing cells (+STAT1-OE), restoring nuclear localization. Co-IP (Fig. 7B) revealed minimal STAT1 lactylation (Klac: 0.014 ± 0.002) and weak Runx2 binding (0.582 ± 0.108) in scaffold-free Runx2-OE cells. STAT1-OE increased binding by 45.2 % (0.845 ± 0.012, *p* < 0.01) but lactylation remained low (0.327 ± 0.046). Lactate treatment in scaffold-containing cells (+STAT1-OE) robustly elevated STAT1 lactylation by 2.4-fold (0.797 ± 0.105, *p* < 0.001), concomitant with a 66.1 % reduction in STAT1-Runx2 binding (0.286 ± 0.050, *p* < 0.001), phenocopying the minimal binding in STAT1-KD cells (0.056 ± 0.011). This establishes lactylation as a metabolic switch regulating STAT1's inhibitory function.

Western blot (Fig. 7C) confirmed consistent Runx2 overexpression across groups. Osteocalcin protein was 3.4-fold higher in Runx2-OE + STAT1-KD versus Runx2-OE alone (0.488 ± 0.075 vs 0.143 ± 0.007; *p* < 0.001), suppressed by 81.8 % in Runx2-OE + STAT1-OE, and rescued to STAT1-KD-like levels (0.474 ± 0.047, *p* < 0.001) in scaffold-containing cells. ChIP-qPCR (Fig. 7D) showed STAT1-KD increased Runx2-promoter binding 1.8-fold versus Runx2-OE (28.811 ± 1.516 vs 15.6-fold; *p* < 0.01). Binding was suppressed by 56.4 % with STAT1-OE (7.321 ± 0.392; *p* < 0.001) but restored 3.3-fold in scaffold-containing cells (24.425 ± 1.076; *p* < 0.001). These data conclusively link STAT1 lactylation to disruption of STAT1-Runx2 binding and subsequent Runx2 transcriptional activation.

### 3.7 Genetic validation of mechanistic necessity

**Fig. 8:**
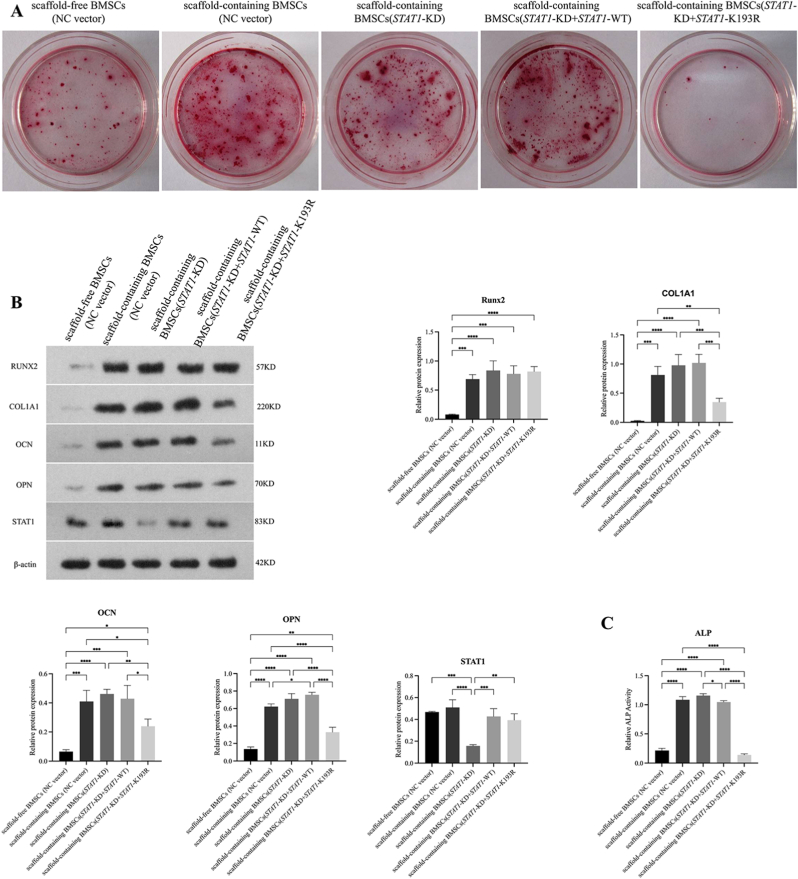
Genetic validation of STAT1 lactylation mechanism. (A) ARS staining (Day 14) showing mineralization differences. (B) Western blot of osteogenic markers and STAT1 (Day 14). (C) ALP activity reduction in STAT1-KD + K193R mutant groups. (Data normalized to β-actin; Data expressed as mean ± SD; ∗*p* < 0.05, ∗*∗p* < 0.01, ∗∗∗*p* < 0.001, ∗∗∗∗*p* < 0.0001).

To definitively validate K193 lactylation's functional necessity, we analyzed five groups: Scaffold-free BMSCs (NC vector), Scaffold-containing BMSCs (NC vector, STAT1-KD, STAT1-KD + STAT1-WT, STAT1-KD + STAT1-K193R). ARS staining (Fig. 8A) revealed stark mineralization contrasts: STAT1-WT rescue in scaffold-containing STAT1-KD cells showed robust calcium deposition, whereas STAT1-K193R rescue exhibited minimal mineralization—comparable to scaffold-free NC controls. Western blot and ALP analyses (Fig. 8B and C) confirmed severely reduced osteogenic markers in STAT1-K193R versus STAT1-WT rescue (COL1A1: 0.348 ± 0.064 vs 1.019 ± 0.147, *p* < 0.01; Osteocalcin: 0.239 ± 0.050 vs 0.429 ± 0.092, *p* < 0.05; OPN: 0.329 ± 0.057 vs 0.757 ± 0.028, *p* < 0.05). STAT1 levels remained equivalent between rescue groups (STAT1-K193R: 0.393 ± 0.059; STAT1-WT: 0.427 ± 0.072; *p* > 0.05). These data unequivocally establish K193 lactylation as the molecular switch controlling STAT1's osteogenic regulation.

### 3.8 *In vivo* bone regeneration

**Fig. 9:**
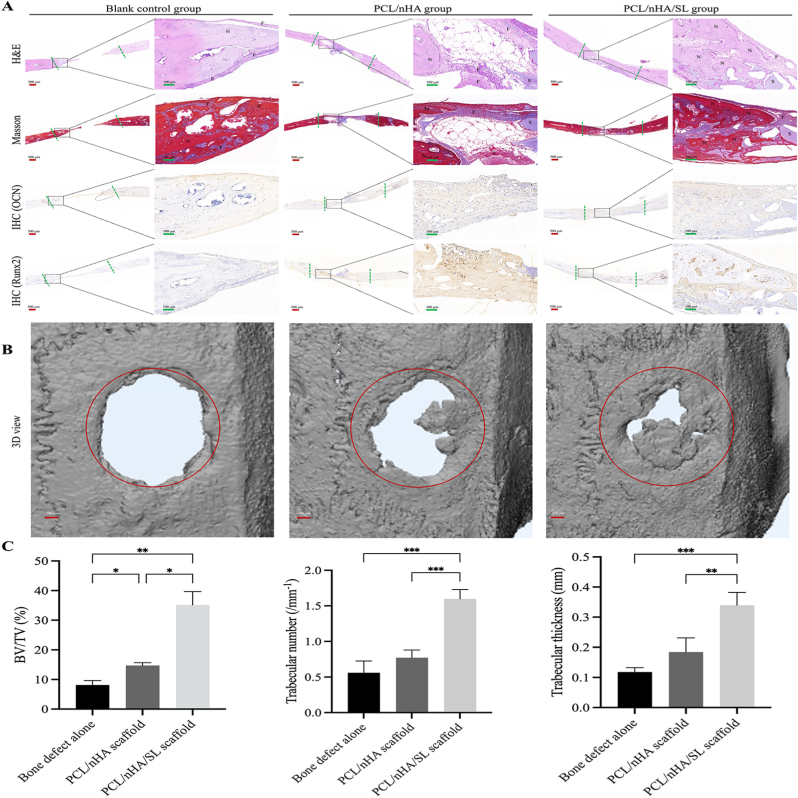
In vivo bone regeneration performance. (A) Histological analysis at 12 weeks: H&E, Masson's trichrome, and IHC for OCN/Runx2 (Red scale bars: 500 μm; green scale bars: 100 μm; N: new bone; F: fibrous tissue; S: residual scaffold). (B) 3D micro-CT reconstruction of defect sites (Red scale bars: 500 μm). (C) Quantitative micro-CT parameters: BV/TV (%), trabecular number (mm^−1^), and thickness (mm). (Data expressed as mean ± SD; ∗*p* < 0.05, ∗*∗p* < 0.01, ∗∗∗*p* < 0.001, ∗∗∗∗*p* < 0.0001). (For interpretation of the references to colour in this figure legend, the reader is referred to the Web version of this article.)

Histological analysis (HE/Masson's; Fig. 9A) at 12 weeks post-implantation showed progressive bone regeneration. The control (bone defect alone) exhibited minimal new bone formation—limited to sparse peripheral trabeculae with central fibrous tissue. The PCL/nHA scaffold group showed enhanced osteogenesis versus control, though incomplete regeneration persisted (residual scaffolds/fibrous tissue in core). Strikingly, the PCL/nHA/SL group achieved near-complete repair with dense, well-organized trabecular networks resembling native bone. Immunohistochemistry corroborated these findings: OCN/RUNX2 staining was weak/localized in control and PCL/nHA groups, but robust and continuous throughout the defect in PCL/nHA/SL, indicating sustained osteogenic activation.

Micro-CT (Fig. 9B and C) quantified outcomes: PCL/nHA/SL exhibited near-complete defect filling with highly interconnected trabeculae, yielding significantly higher BV/TV, Tb.N, and Tb.Th (*p* < 0.01 vs. control; *p* < 0.05 vs. PCL/nHA). In contrast, PCL/nHA showed partial deposition with residual voids, while the control displayed only peripheral disorganized trabeculae. These structural improvements mirror our prior report where lactate metabolism amplified epigenetic reprogramming to accelerate mineralization.

## 4 Discussion

The development of lactate-functionalized 3D-printed PCL/nHA/SL scaffolds presents a promising strategy to address metabolic challenges in bone defect repair under normoxic conditions. We demonstrate that sodium lactate modification restores hMSC osteogenic potential through dual metabolic-epigenetic mechanisms, offering a biomaterial solution integrating structural support with biochemical modulation.

Human MSCs naturally reside in hypoxic bone marrow niches, where glycolytic metabolism maintains their quiescence and multipotency [22]. Conventional in vitro expansion and subsequent implantation into normoxic environments disrupt this metabolic equilibrium, shifting cells toward oxidative phosphorylation and accelerating mitochondrial dysfunction and senescence [23]. Our PCL/nHA/SL scaffolds mitigate these adverse effects by providing sustained lactate release—a key glycolytic byproduct. Beyond serving as an alternative energy substrate, lactate stabilizes hypoxia-inducible factor 1α (HIF-1α), thereby mimicking hypoxic signaling to preserve hMSC stemness [24,25]. Critically, the controlled degradation kinetics of the scaffolds ensure prolonged lactate availability aligned with the bone healing timeline. Throughout the 28-day period, the microenvironment maintained mild acidity (pH 5.8–6.6) and continuous low-concentration Ca^2+^ release from nHA. This deliberately orchestrated acidic milieu synergizes with calcium ions to promote osteogenesis while preventing acidosis [26,27], paralleling reports that lactylation directly regulates osteogenic transcription—as evidenced by proanthocyanidin-mediated rescue of LPS-inhibited osteogenesis via lysine lactylation restoration in PDLSCs [28]. Our approach thus converges with strategies utilizing hypoxia mimetics or 3D culture systems for metabolic preconditioning [29,30], underscoring metabolic-epigenetic coordination in tissue engineering.

Beyond metabolism, lactate regulates osteogenesis through lysine lactylation (K-Lac), a post-translational modification with epigenetic implications [31,32]. Our proteomics identified STAT1-K193 lactylation triggers its cytoplasmic translocation, disrupting inhibitory binding to Runx2 and liberating Runx2 to drive osteogenic transcription. This represents a paradigm shift from established mechanisms: 1) Unlike SOCS1-mediated STAT1/3 inhibition that suppresses osteogenesis [33], lactylation redistributes STAT1 spatially without altering phosphorylation; 2) Whereas JAK2 inhibitors (fedratinib) and metformin impair osteogenesis via JAK-STAT suppression [34,35], lactylation bypasses kinase-dependent activation; 3) Contrasting immunomodulatory approaches that indirectly influence osteogenesis through macrophage polarization [36], our mechanism directly converts glycolytic flux into epigenetic instructions via post-translational modification. This evolutionarily conserved process establishes lactylation as a metabolic-epigenetic rheostat - repurposing STAT1 from transcriptional repressor to cytoplasmic sequestered protein, thereby resolving the STAT1-Runx2 conflict during normoxic stress. By creating a permissive chromatin landscape through direct protein retargeting rather than canonical pathway inhibition, this mechanism offers a novel resolution to metabolic-epigenetic crosstalk in bone regeneration.

Lactate's immunomodulatory roles in bone regeneration warrant specific consideration. Emerging evidence demonstrates that lactate directly reprograms macrophage function through metabolic and signaling pathways: (1) It sustains efferocytosis capacity by enhancing PFKFB2-mediated glycolysis, enabling macrophages to continuously clear apoptotic cells during inflammation resolution [37]; (2) It suppresses pro-inflammatory responses via GPR81-dependent inhibition of YAP/NF-κB activation [38]; and (3) It accelerates anti-inflammatory M2 polarization through STAT3 phosphorylation, promoting tissue repair as validated in myocardial infarction models [16]. These mechanisms align with the immunometabolic switch observed during early bone healing. While our scaffold's sustained lactate release may synergize with these pathways—particularly in modulating the initial inflammatory phase—direct experimental validation of lactate-immune cell crosstalk within our system remains unexplored. Future studies should validate whether scaffold-released lactate reproduces its immunomodulatory effects on macrophages observed in other systems, addressing this key knowledge gap.

Traditional bone grafts, such as PCL/nHA composites, often exhibit limited efficacy due to static architectures and poor adaptation to dynamic metabolic healing phases [39,40]. In contrast, our SL-reinforced PCL/nHA scaffold provides biphasic functionality: nHA delivers osteoconductive support and mineral ion release, while spatiotemporal lactate delivery—governed by PCL degradation—enables continuous metabolic reprogramming and lactylation-dependent signaling. This design recapitulates the natural bone repair sequence (early inflammation/angiogenesis followed by mineralization). Furthermore, scaffold degradability ensures temporal alignment between lactate availability and regenerative processes, including neutrophil-mediated mobilization and immunomodulation, without inducing adverse immune effects [15,18,38]. Nevertheless, as noted above, explicit characterization of lactate-driven immune cell regulation is lacking. Compared to growth factor-loaded scaffolds or cell-based therapies [41,42], our approach leverages endogenous hMSC recruitment via localized biochemical cues, reducing risks of ectopic ossification while enhancing translational feasibility.

From a translational perspective, **3D printing** enables patient-specific designs adaptable to defect geometry [43]. Future systems could incorporate real-time biomarkers (e.g., lactate sensors) to dynamically adjust drug release kinetics [5], advancing toward adaptive implants for precision medicine. Several considerations warrant further investigation: 1) Systematic evaluation of scaffold-derived lactate on immune cells (e.g., macrophage polarization in contaminated defects where excessive anti-inflammatory signaling might compromise infection control [44]); 2) Personalized optimization of lactate release profiles to accommodate variations in defect size, vascularity, and metabolic status; 3) Long-term assessment of scaffold resorption and confirmation that sustained lactate exposure preserves extracellular matrix homeostasis.

Collectively, these findings establish PCL/nHA/SL scaffolds as a multifunctional platform addressing structural and metabolic barriers in bone regeneration. By synergizing material design with stem cell biology, this work demonstrates how biomaterials can be engineered to interface with cellular biochemistry, providing a roadmap for next-generation regenerative therapies.

## 5 Conclusion

This study establishes **PCL/nHA/SL scaffolds** as a multifunctional platform enhancing bone regeneration via **STAT1 lactylation-mediated Runx2 activation**. By synergizing osteoconductive nHA with lactate-driven epigenetic modulation, our design overcomes limitations of static grafts and growth factor dependency. While significant in vivo defect repair is demonstrated, future studies should optimize lactate release kinetics, characterize immune microenvironment crosstalk, and validate long-term scaffold resorption. These findings highlight the promise of metabolically active biomaterials for next-generation tissue engineering.

## CRediT authorship contribution statement

**Min Zeng:** Writing – original draft, Visualization, Methodology, Investigation, Formal analysis, Data curation, Conceptualization. **Hao Liu:** Investigation, Formal analysis. **Wei Lu:** Validation, Investigation. **Can Chen:** Methodology, Formal analysis. **Zhangyuan Lin:** Supervision, Investigation. **Ruibo Zhao:** Writing – review & editing, Supervision, Project administration, Funding acquisition.

## Funding

This work was supported by the 10.13039/501100004735Natural Science Foundation of Hunan Province, China (No. 2022JJ40768, 2022SK2027).

## Declaration of competing interest

The authors declare that they have no known competing financial interests or personal relationships that could have appeared to influence the work reported in this paper.

## Data Availability

Data will be made available on request.
